# Etiology and Outcomes of Hepatocellular Carcinoma in an Ethnically Diverse Population: The Multiethnic Cohort

**DOI:** 10.3390/cancers13143476

**Published:** 2021-07-12

**Authors:** Afsaneh Barzi, Kali Zhou, Songren Wang, Jennifer L. Dodge, Anthony El-Khoueiry, Veronica Wendy Setiawan

**Affiliations:** 1 City of Hope Comprehensive Cancer Center, Department of Medical Oncology, Duarte, CA 91010, USA; afsaneh.barzi@myaccesshope.org; 2 Division of Gastrointestinal and Liver Diseases, Department of Medicine, Keck School of Medicine of University of Southern California, Los Angeles, CA 90033, USA; Kali.Zhou@med.usc.edu (K.Z.); Jennifer.Dodge@med.usc.edu (J.L.D.); 3 Department of Population and Public Health Sciences, Keck School of Medicine of University of Southern California, Los Angeles, CA 90033, USA; songrenw@usc.edu; 4 Norris Comprehensive Cancer Center, Keck School of Medicine of University of Southern California, Los Angeles, CA 90033, USA; elkhouei@usc.edu

**Keywords:** liver cancer, ethnicity, racial disparities, HCV, steatosis, NAFLD, medicare

## Abstract

**Simple Summary:**

Racial/ethnic disparities in the incidence and outcomes of hepatocellular carcinoma (HCC) is previously described. Yet, due to the challenges for ascertainment of the underlying etiology (e.g., hepatitis B and hepatitis C) in registry-based studies, the contribution of the underlying etiology to the racial disparities is poorly described. Utilizing comprehensive data on tumor characteristics, lifestyle, and outcomes in the Multiethnic Cohort Study, we explored racial disparities in HCC. We show significant racial disparities in the underlying etiology, mortality, and treatment patterns. We further show that underlying etiology is a significant contributor to racial disparities in mortality by race and should be considered in future research.

**Abstract:**

Backgrounds: HCC incidence varies by race/ethnicity. We characterized racial differences in underlying etiology, presentation, and survival in the linkage of Multiethnic Cohort Study with SEER and Medicare claims. Methods: HCC characteristics, treatment, and underlying etiology in participants were obtained. Deaths were ascertained using state death certificates and the National Death Index. Risk factors were collected via questionnaires. Cox models were used to calculate hazard ratios (HR) and 95% confidence intervals (CI) for death. Results: Among 359 cases, the average age at diagnosis was 75.1. The most common etiology was hepatitis C (HCV) (33%), followed by nonalcoholic fatty liver disease (NAFLD) (31%), and different by ethnicity (*p* < 0.0001). African Americans (AA) (59.5%) and Latinos (40.6%) were more likely to be diagnosed with HCV-related HCC. In Japanese Americans (33.1%), Native Hawaiians (39.1%), and whites (34.8%), NAFLD was the most common etiology. Receipt of treatment varied across ethnic groups (*p* = 0.0005); AA had the highest proportion of no treatment (50.0%), followed by Latinos (45.3%), vs. whites (15.2%). HCC (72.2%) was the most common cause of death. In a multivariate analysis, AA (HR = 1.87; 95% CI: 1.06–3.28) had significantly higher mortality compared to whites. Conclusions: We found significant ethnic differences in HCC underlying etiology, receipt of treatment, and outcome. The findings are important for reducing disparities.

## 1. Introduction

Despite declining incidence and mortality of several cancers in the United States and worldwide, the incidence and mortality of hepatocellular carcinoma (HCC) are on the rise [1,2,3,4,5]. Minorities are disproportionately affected by HCC, with highest incidence seen among Hispanics and Asians [3,6,7]. In the Multiethnic Cohort Study (MEC), a large prospective and ethnically diverse cohort with long-term follow-up, we previously reported the highest HCC incidence in Latinos, followed by Native Hawaiians, Japanese Americans, African Americans, and whites [8,9]. Studies have suggested notable racial/ethnic disparities in HCC survival as well, with lower survival among African Americans and Hispanics, independent of tumor stage, treatment, and socioeconomic status [6,10,11,12,13].

HCC is rare in individuals with normal liver and is contingent on liver injury from a variety of different etiologies. The most common etiologies include infectious hepatitis (hepatitis B and C), high alcohol consumption, and non-alcoholic fatty liver disease (NAFLD). Distribution of underlying etiology of liver disease varies substantially by race/ethnicity and may dictate the risk of development, detection, and outcome of HCC [1,14,15,16]. For example, hepatitis B (HBV) is endemic in parts of Asia and sub-Saharan Africa, and the highest rates of HBV infection in the USA are seen among immigrants from highly endemic countries [17]. Hepatitis C (HCV) is more common among black than white Americans, as well as Asian ethnic subgroups such as Japanese [18,19]. NAFLD and its most severe form non-alcoholic steatohepatitis (NASH) are rapidly becoming the most common cause of chronic liver disease in the United States and disproportionately impact Hispanics [14,20]. Yet, the impact of underlying etiological factors in conjunction with race/ethnicity on HCC outcome is not well explored.

Studies on racial/ethnic disparities in HCC survival largely rely on cancer registries, which lack information about underlying liver disease and risk factors, thus providing us an incomplete understanding of why these disparities exist [3,6,21]. Many studies have described survival disparities by race/ethnicity, particularly higher mortality among African Americans, although few incorporated liver disease etiology and most are retrospective in nature [12,13,16,22]. The prospective and population-based MEC is an ideal study to evaluate underlying etiology of HCC in conjunction with race/ethnicity. MEC not only contains detailed information about individual-level demographic and HCC risk factors, such as alcohol intake, obesity, and diabetes mellitus, but it is also enriched with cancer registry data on tumor characteristics, Medicare claims, and mortality information. Here, we provide an in-depth analysis of HCC characteristics, underlying chronic liver disease etiology, receipt of treatment, and outcome in an ethnically diverse population.

## 2. Materials and Methods

### 2.1. Study Population

We conducted a prospective analysis of HCC in the MEC. Briefly, the MEC is a large prospective cohort with >215,000 men and women aged 45–75 years, living in Hawaii and California at cohort entry (1993–1996). The cohort participants have been followed for more than two decades. Cohort design and baseline characteristics have been previously described [9]. The baseline mailed questionnaire assessed demographic; diet; lifestyle; anthropometry; family and personal medical history; and, for women, menstrual and reproductive history and hormone use. Annual linkages with the statewide Surveillance, Epidemiology, and End Results Program (SEER) registries of Hawaii and California were used to identify incident cases of cancer. Cause and date of death were ascertained using linkages with death certificate files for Hawaii and California and the National Death Index. The Institutional Review Boards for the University of Southern California (HS-17-00714) and the University of Hawaii (CHS 9575) approved this study.

### 2.2. HCC Identification and Tumor Characteristics

A total of 742 HCC incident cases (code C22.0 and morphology codes 8170–8175) were identified via SEER linkage between cohort entry and 2014. Tumor characteristics (stage at diagnosis and histology), year of diagnosis, and initial treatment modality information were obtained from SEER. We restricted the study population to a subset of HCC cases (*n* = 359) who were enrolled in the Medicare fee-for-service (FFS) because we utilized Medicare claims to identify HCC underlying etiology. The characteristics between excluded cases and those included in the study were similar with respect to sex, body mass index (BMI), smoking status, education, and cancer stage (Table 1 and Table S1). As expected in a Medicare population, included cases had older age at diagnosis and were more likely to have received treatment. As previously described [23], we identified underlying HCC etiology using 1 inpatient or ≥2 outpatient/carrier claims using International Classification of Diseases (ICD) 9th and 10th revision codes. We followed a published set of diagnostic criteria [24] to define the underlying HCC etiology [23]. Using ICD codes, we identified hepatitis B and C infection, alcohol-related liver disease (ALD), NAFLD, and other causes of liver disease (e.g., hemochromatosis, primary biliary cirrhosis, primary sclerosing cholangitis, Wilson’s disease, human immunodeficiency virus (HIV), alpha-1-antitrypsin deficiency, and autoimmune hepatitis). NAFLD was defined as the underlying etiology based on ICD codes (ICD-9: 571.8 and 571.9; ICD-10: K75.81, K760, K7689, K741, K769), along with diabetes and BMI information and after excluding other underlying etiology listed above. Following the AASLD guidelines [25], cases who reported >21 drinks/week (men) or >14 drinks/week (women) were reclassified as ALD-related HCC. Cases who did not meet any of the above criteria were classified as cryptogenic and grouped with other etiology. Disease severity including the presence of hepatic encephalopathy (ICD-9: 572.2 and ICD-10 code K72.90, K72.91), ascites (ICD-9: 789.51, 789.59, and ICD-10 code R18.0, R18.8), or esophageal varices (ICD-9: 456.0, 456.1, 456.2 and ICD-10 code I85.00, I85.01, I85.10, I85.11) was determined using Medicare claims prior to HCC diagnosis.

Covariates: Information about education, BMI (kg/m^2^), smoking status, alcohol intake, and diet were obtained from baseline questionnaire. To capture aspects of the entire diet and to better examine the complexity of foods and beverages as consumed, we utilized the Healthy Eating Index-2010 (HEI-2010) score (higher scores reflect better diet quality), which has been associated with liver diseases and HCC in previous studies [26,27]. Diabetes mellitus was assessed via baseline questionnaire and Medicare claims as previously described [28].

### 2.3. Statistical Analysis

Fisher’s exact test and the chi-squared test were used to compare tumor characteristics and risk factors by race/ethnicity. Our main analysis was for all-cause mortality, but we also examined HCC specific mortality (ICD-O-3 code: C220, C229) and other liver-related mortality (ICD-10 codes: B171, B182, B942, K703, K729, K743, K746, K769). Kaplan–Meier survival was calculated from date of HCC diagnosis to death, censoring participants remaining alive at the end of the follow up period (31 December 2014) and compared by race/ethnicity using the log-rank test. As there were 10 pairwise comparisons, we presented the overall *p*-value for the equality over strata where significance indicated at least one stratum differed from another. Univariate and multivariate Cox proportional hazard models were used to calculate hazard ratios (HR) and 95% confidence intervals (CI) for associations between race/ethnicity, sex, tumor characteristics, treatment, and risk factors with all-cause and HCC mortality. All tests were two-sided. Statistical analyses were performed using SAS 9.4 (SAS, Cary, NC, USA).

## 3. Results

### 3.1. Cohort Characteristics by Race/Ethnicity

The study population consisted of 359 incident HCC cases (Table 1). The largest racial/ethnic group represented was Japanese Americans (*n* = 142; 40%), followed by Latinos (*n* = 106; 29%), whites (*n* = 46; 13%), African Americans (*n* = 42; 12%), and lastly Native Hawaiians (*n* = 23; 6%). A total of 66% of HCC cases were male. Ever smoking was common among HCC cases (71%), and the prevalence of ever smokers differed across ethnic groups; highest in African Americans (81%) and lowest in Latinos (62%). The prevalence of obesity (BMI ≥ 30 kg/m^2^) was also different across racial/ethnic groups; whites had the highest prevalence of obesity (37%) compared to other populations, and Japanese Americans had the lowest prevalence (18%). The prevalence of diabetes was high in this population (62%); the highest prevalence was observed in Latinos (75%). High daily intake of alcohol (≥12 ethanol g/day) was reported in 23% of HCC cases. Daily use of alcohol was highest in Native Hawaiians (52%) and lowest in Japanese Americans (17%). The highest HEI-2010 score, indicating better diet quality, was observed in whites. The lowest median score was observed in Latinos.

### 3.2. HCC Characteristics and Underlying Etiology by Race/Ethnicity

**Figure 1:**
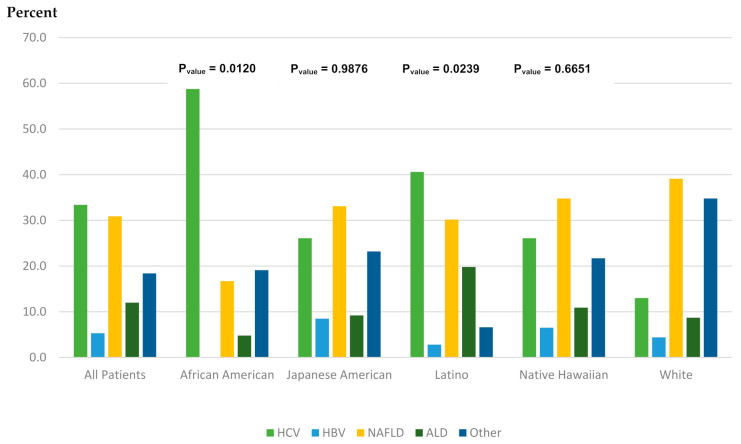
Underlying etiology overall and by racial/ethnic groups. *p*-value comparing each ethnic group to whites.

Median age at diagnosis of HCC was 75.1 years (ranging from 72.9 in whites to 76.7 in African Americans) (Table 2). Most cases were diagnosed with localized stage (47%) with no significant difference in the stage distribution by race/ethnicity (*p* = 0.76). The majority of HCC cases received treatment (61%), higher among whites (72%) and Japanese Americans (68%) and lower in Latinos (51%) and African Americans (48%). In all ethnic groups combined, the most common underlying etiology for HCC was HCV (33%) followed by NAFLD (31%), other including cryptogenic (18%), ALD (12%), and HBV (5%) (Figure 1). There were significant differences in the distribution of underlying etiology across racial/ethnic groups (*p* < 0.0001). HCV was the most common underlying etiology in Latinos (40.6%) and African Americans (59.5%), while NAFLD was the most common etiology in whites (34.8%), Japanese Americans (33.1%), and Native Hawaiians (39.1%). HCV was the second most common etiology in Japanese Americans (26.1%) and whites (26.1%).

### 3.3. Relationship between Race/Ethnicity, Risk Factor, and Mortality

Of 359 HCC cases, 295 (82%) were deceased (Table 2). HCC was the most common cause of death (72%), followed by non-liver-related deaths (20%) and liver-related deaths (8%). HCC was the most common cause of death in each racial/ethnic group (ranging from 69% in Japanese Americans to 83% in Native Hawaiians). Median follow-up time in whites was 14.7 months (median survival 11 months), Japanese Americans—12.4 months (median survival 6.3 months), Native Hawaiians—12.2 months (median survival 2.3 months), Latinos—8.8 months (median survival 8.2 months), and African Americans—6.4 months (median survival 5.7 months).

**Figure 2:**
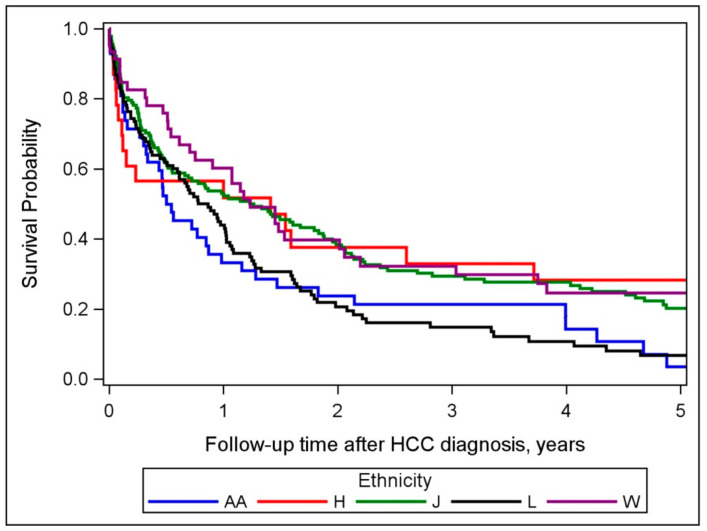
Overall (all-cause mortality) survival by racial/ethnic groups (Log-rank *p*-value = 0.0126). Abbreviations: B = blacks; H = Native Hawaiians; J = Japanese Americans; L = Latinos; W = whites.

We observed a significant difference in survival by race/ethnicity (*p* = 0.0126), with the lowest rate in Latinos and African Americans (Figure 2). Factors associated with all-cause mortality are presented in Table 3. In univariate analysis, race/ethnicity, underlying etiology, cancer stage, year of diagnosis, treatment status, smoking, education, BMI of more than 25 kg/m^2^, and HEI-2010 were all significantly associated with mortality. Sex, alcohol intake, diabetes mellitus, and disease severity were not associated with mortality. For race/ethnicity, African Americans and Latinos with HCC had higher mortality risks compared to whites. In multivariate analysis, in addition to stage and treatment, race/ethnicity, obesity (BMI greater than 30), smoking status, and underlying etiology remained independent predictors of overall mortality. Compared to HCV-related HCC, NAFLD- (HR = 2.02; 95% CI: 1.40, 2.91), and ALD (HR = 1.73; 95% CI: 1.11, 2.72) were associated with higher mortality risk (Figure S1). Obesity (HR = 1.89; 95% CI: 1.29, 2.75) and smoking status (HR = 1.72; 95% CI: 1.26, 2.33) were associated with higher mortality, while higher education was associated with lower mortality (HR for some college vs. high school = 0.72; 95% CI: 0.52, 0.98). African Americans (HR = 1.87; 95% CI: 1.06, 3.28) had significantly higher mortality, while Latinos, Japanese Americans, and Native Hawaiians had no significant differences in mortality compared to whites. Similar results were observed when analysis was resricted to HCC mortality (Table S2).

## 4. Discussion

In the MEC, we found significant racial/ethnic differences in HCC underlying etiology, receipt of treatment, and disease outcome. Stage of cancer and receipt of treatment are undoubtedly important predictors of HCC survival. We demonstrate that HCC underlying etiology, smoking status, and obesity are independent predictors of overall mortality in patients with HCC. African Americans with HCC had higher mortality compared to whites after adjusting for tumor characteristics, treatment and these additional risk factors.

The MEC cohort is unique in its enrichment for minorities, making it an ideal cohort for assessing the impact of race/ethnicity on HCC survival. Additionally, the interplay between race/ethnicity, underlying liver disease etiology, and survival remains understudied, and our analysis provides an insight into these interactions. Previous studies have used SEER data or other cancer registries to assess the impact of race on the diagnosis and survival of HCC [13,29]; while these registries are a major source for understanding the fundamentals of disparities in cancer patients and various populations, HCC’s dependence on an underlying liver disease and other risk factors poses a significant challenge. Without considering etiology, these studies showed that African American race was a significant predictor of poor HCC survival, including among those with early stage disease and/or within criteria for liver transplantation. The conglomeration of several underlying etiologies in different racial groups questions the independent role of race as a predictor of survival. Here, we show that HCV-related HCC is associated with better survival (*p* = 0.0019) (Figure S1), and with inclusion of the underlying etiology, race remains an independent predictor of HCC survival. While differential access to treatment may play a role in the racial disparity of survival, SEER-UNOS (United Network for Organ Sharing) data showed that survival of African Americans remained worse than whites after liver transplantation for HCC [10].

Furthermore, the difference in survival by underlying etiology (specifically, viral vs. metabolic) was also described in a recent SEER-Medicare publication [30]. It is possible that the underlying etiology drives the natural history of HCC and candidacy for treatment, e.g., frequency of non-cirrhotic HCC differs by etiology [31]. Yet, patterns of care play an important role in the observed differences between viral vs. other etiologies of HCC. Due to national recommendations for HBV and HCV screening, patients with these etiologies may be more likely to be in specialist care, and therefore more likely to receive higher quality care for their HCC by a multidisciplinary team of specialists. Lack of information on surveillance history, underlying liver function, and specific therapeutic interventions limits our ability to make further conclusions.

Our data did show a significant racial/ethnic disparity in receipt of treatment, as well as the fact that both African Americans and Latinos have a higher likelihood of going untreated. Socioeconomic factors, such as neighborhood deprivation, poverty, and lack of insurance, have been hypothesized as reasons for lower cancer survival among African Americans [32]. Given that this analysis is restricted to MEC-Medicare participants, it is presumed that these patients are insured, and thus insurance alone cannot account for the survival disparity as evidenced by the lower rates of no treatment in the HCC cases in Medicare than excluded cases (Table S1). Reasons for no treatment in minorities need to be further explored. Medical mistrust is well documented in the African American community but has not been explored with respect to HCC treatment; conversely, implicit bias may lead to lower frequency of treatment offered [33,34]. Furthermore, the severity of liver dysfunction is not captured in this study and reduced eligibility for treatment due to more decompensated cirrhosis in these patients might be a key reason. Given the strong evidence for HCC disparities in African Americans, future studies need to focus on elucidating the root determinants, particularly surrounding lower treatment uptake, of such disparities to inform effective interventions.

Our study has several strengths including its prospective design, multiethnic population, and availability of personal demographic and HCC risk factors (i.e., obesity, diabetes, alcohol intake, smoking, etc.) that are not available from cancer registries. Our study also has several limitations, including treatment data obtained from registry linkage that is not as detailed as those from clinical studies, and given the unique treatment paradigms of HCC, inaccuracy reporting of treatment in registry is possibile [35]. Information on liver function, an important determinant of HCC outcome, was not available in this study. Furthermore, etiology identification was based on Medicare claims that can lead to misclassification of disease etiology. However, because we utilized questionnaire data to refine classification and used the same methods across racial/ethnic groups, the misclassification should be similar across groups. Another limitation is that our study size was relatively small compared to registry-based studies and included older Medicare-FFS population which limits generalizability to other populations.

## 5. Conclusions

We identified significant racial/ethnic differences in HCC underlying etiology and disease outcomes beyond the impact of underlying etiology. Acknowledging differences in underlying HCC etiology and access to treatment in different racial/ethnic groups is important for improving HCC outcomes and reducing disparities.

## Figures and Tables

**Table 1:** Baseline characteristics of HCC in the Multiethnic Cohort by race/ethnicity.

Characteristics	All Patients (*N* = 359)	African American (*N* = 42)	Japanese American (*N* = 142)	Latino (*N* = 106)	White (*N* = 46)	Native Hawaiian (*N* = 23)
Mean age at enrollment (SD), years	61.5 ± 7.43	64.1 ± 7.65	62.5 ± 7.75	60.2 ± 5.94	59.6 ± 7.91	60.7 ± 8.61
Sex (%)						
Male	235 (65.5)	27 (64.3)	71 (62.0)	88 (67.0)	32 (69.6)	17 (73.9)
Female	124 (34.5)	15 (35.7)	54 (38.0)	35 (33.0)	14 (30.4)	6 (26.1)
BMI category (%)						
<25.0 kg/m^2^	106 (29.5)	12 (28.6)	57 (40.1)	19 (17.9)	12 (26.1)	17 (26.1)
25.0–30.0 kg/m^2^	155 (43.2)	19 (45.2)	58 (40.9)	51 (48.1)	17 (37.0)	10 (43.5)
≥30.0 kg/m^2^	95 (26.5)	9 (21.4)	26 (18.3)	36 (34.0)	17 (37.0)	7 (30.4)
Unknown	3 (0.8)	2 (4.8)	1 (0.7)	0 (0.0)	0 (0.0)	0 (0.0)
Smoking status (%)						
Never smoker	96 (26.7)	8 (19.1)	38 (26.8)	35 (33.0)	12 (26.1)	3 (13.0)
Ever smoker	255 (71.1)	34 (80.9)	104 (73.2)	66 (62.3)	33 (71.7)	18 (78.3)
Unknown	8 (2.2)	0 (0.0)	0 (0.0)	5 (4.7)	1 (2.2)	2 (8.7)
Education (%)						
High school graduate or less	170 (47.4)	20 (47.6)	58 (40.9)	72 (67.9)	10 (21.7)	10 (43.5)
Some college/technical school	112 (31.2)	15 (35.7)	47 (33.1)	21 (19.8)	20 (43.5)	9 (39.1)
College graduate	35 (9.8)	5 (11.9)	15 (10.6)	5 (4.7)	8 (17.4)	2 (8.7)
Graduate/professional school	34 (9.5)	2 (4.8)	21 (14.8)	3 (2.8)	7 (15.2)	1 (4.4)
Unknown	8 (2.2)	0 (0.0)	1 (0.7)	5 (4.7)	1 (2.2)	1 (4.4)
Alcohol Intake (%)						
0	176 (49.0)	19 (45.2)	82 (57.8)	49 (46.2)	18 (39.1)	8 (34.8)
<12 g/day	83 (23.1)	11 (26.2)	30 (21.1)	28 (26.4)	12 (26.1)	2 (8.7)
≥12 g/day	84 (23.4)	11 (26.2)	24 (16.9)	23 (21.7)	14 (30.4)	12 (52.2)
Missing	16 (4.5)	1 (2.4)	6 (4.2)	6 (5.7)	2 (4.4)	1 (4.4)
Diabetes mellitus (%)	223 (62.1)	21 (50.0)	89 (62.7)	79 (74.5)	22 (47.8)	12 (52.2)
Healthy Eating Index 2010						
Median (range)	64.5 (32.1–93.7)	66.5 (32.1–85.6)	64.6 (34.6–86.8)	62.8 (45.0–92.3)	69.5 (41.0–93.7)	67.8 (51.0–78.8)

**Table 2:** Tumor characteristics of HCC and deaths in the Multiethnic Cohort by race/ethnicity.

Characteristics	All Patients (*N* = 359)	African American (*N* = 42)	Japanese American (*N* = 142)	Latino (*N* = 106)	White (*N* = 46)	Native Hawaiian (*N* = 23)
Mean age at diagnosis (SD), years	75.1 ± 7.16	76.7 ± 8.26	76.2 ± 7.40	74.0 ± 5.86	72.9 ± 7.27	74.7 ± 7.47
Median follow-up time *, months	10.6	6.4	12.4	8.8	14.7	12.2
Stage at diagnosis (%)						
Localized	169 (47.1)	18 (40.5)	72 (50.7)	51 (48.1)	21 (45.7)	8 (34.8)
Regional	87 (24.2)	11 (26.2)	37 (26.1)	24 (22.6)	10 (21.7)	5 (21.7)
Distant	57 (15.9)	7 (16.7)	22 (15.5)	15 (14.2)	8 (17.4)	5 (21.7)
Unknown	46 (12.8)	7 (16.7)	11 (7.8)	16 (15.1)	7 (15.2)	5 (21.7)
*p*-value **	0.7605	0.9495	0.4677	0.9645	Reference	0.7741
Underlying Etiology						
HCV	120 (33.4)	25 (59.5)	37 (26.1)	43 (40.6)	12 (26.1)	3 (13.0)
HBV	19 (5.3)	0 (0.0)	12 (8.5)	3 (2.8)	3 (6.5)	1 (4.4)
NAFLD	111 (30.9)	7 (16.7)	47 (33.1)	32 (30.2)	16 (34.8)	7 (39.1)
ALD	43 (12.0)	2 (4.8)	13 (9.2)	21 (19.8)	5 (10.9)	2 (8.7)
Other	66 (18.4)	8 (19.1)	33 (23.2)	7 (6.6)	10 (21.7)	8 (34.8)
*p*-value **	<0.0001	0.0120	0.9876	0.0239	Reference	0.6651
Treatment (%)						
None	119 (33.2)	21 (50.0)	37 (26.1)	48 (45.3)	7 (15.2)	6 (26.1)
Treated	218 (60.7)	20 (47.6)	97 (68.3)	54 (50.9)	33 (71.7)	14 (60.9)
Unknown	22 (6.1)	1 (2.4)	8 (5.6)	4 (3.8)	6 (13.0)	3 (13.0)
*p*-value **	0.0005	0.0009	0.1128	0.0007	Reference	0.5387
Conditions (%) ***						
Yes	51 (14.2)	28 (4.8)	21 (14.8)	25 (23.6)	1 (2.2)	2 (8.7)
*p*-value **	0.0021	0.6039	0.0207	0.0013	Reference	0.2559
Cause of death (%)						
All death (n)	295	38	111	93	35	18
HCC-related	213 (72.2)	28 (73.7)	77 (69.4)	68 (73.1)	25 (71.4)	15 (83.3)
Liver-related	22 (7.5)	2 (5.3)	6 (5.4)	12 (12.9)	2 (5.7)	0 (0.0)
Non-liver-related	58 (19.7)	8 (21.1)	27 (24.3)	12 (12.9)	8 (22.9)	3 (16.7)
Unknown	2 (0.7)	0 (0.0)	1 (0.9)	1 (1.1)	0 (0.0)	0 (0.0)

* From HCC diagnosis to death or end of follow up. ** For all patients, *p*-values for differences by race/ethnicity. For each ethnic group, *p*-values for comparing each group to whites. *p*-values from chi-squared test or Fisher’s exact test when cells have expected counts less than 5. *** The presence of ascites, esophageal varices, or hepatic encephalopathy.

**Table 3:** Association of race/ethnicity and other factors with overall mortality.

	No. Deaths	Univariate HR (95% CI) ^a^	*p*-Value	Multivariate HR (95% CI) ^b^	*p*-Value
Race/ethnicity					
White	35	1.00	<0.0001	1.00	0.0067
African American	38	1.73 (1.08–2.78)		1.87 (1.06–3.28)	
Japanese American	111	0.96 (0.65–1.41)		1.02 (0.66–1.57)	
Latino	93	1.92 (1.29–2.85)		1.47 (0.91–2.38)	
Native Hawaiian	18	0.75 (0.42–1.34)		0.65 (0.34–1.23)	
Sex					
Female	105	1.00	0.5638		
Male	190	1.08 (0.84–1.37)			
Underlying etiology					
HCV	96	1.00	0.0007	1.00	0.0030
HBV	14	0.90 (0.51–1.60)		2.00 (1.03–3.86)	
NAFLD	90	1.53 (1.13–2.08)		2.02 (1.40–2.91)	
ALD	40	2.11 (1.44–3.09)		1.73 (1.11–2.72)	
Other	55	1.20 (0.86–1.68)		1.32 (0.88–1.99)	
Stage at diagnosis					
Localized	121	1.00	<0.0001	1.00	<0.0001
Regional	76	2.33 (1.74–3.12)		2.16 (1.57–2.98)	
Distant	56	3.79 (2.74–5.25)		3.06 (2.13–4.39)	
Unknown	42	4.76 (3.29–6.87)		1.76 (1.11–2.78)	
First course of treatment					
None	113	1.00	<0.0001	1.00	<0.0001
Treated	162	0.17 (0.13–0.22)		0.22 (0.16–0.30)	
Unknown	20	1.04 (0.62–1.77)		1.06 (0.57–1.99)	
Smoking status					
Never smoker	75	1.00	0.0238	1.00	0.0021
Ever smoker	213	1.45 (1.11–1.90)		1.72 (1.26–2.33)	
Unknown	7	1.44 (0.66–3.15)		0.98 (0.38–2.51)	
Education					
High school graduate or less	149	1.00	0.0072	1.00	0.1614
Some college/technical school	91	0.82 (0.63–1.07)		0.72 (0.52–0.98)	
College graduate	26	0.90 (0.59–1.38)		0.90 (0.55–1.47)	
Graduate/professional school	22	0.43 (0.27–0.68)		0.63 (0.36–1.09)	
Unknown	7	1.04 (0.48–2.25)		1.43 (0.51–3.99)	
Alcohol intake					
0	145	1.00	0.2200		
<12 g/day	72	1.21 (0.91–1.62)			
≥12 g/day	66	0.93 (0.69–1.25)			
Unknown	12	1.56 (0.85–2.87)			
BMI category (kg/m^2^)					
<25.0	87	1.00	<0.0001	1.00	0.0011
25.0–30.0	124	1.43 (1.08–1.89)		0.98 (0.71–1.36)	
≥30.0	81	2.55 (1.86–3.49)		1.89 (1.29–2.75)	
Unknown	3	1.69 (0.51–5.64)		0.82 (0.22–3.04)	
Diabetes mellitus					
No	112	1.00	0.4653		
Yes	183	1.09 (0.86–1.39)			
Healthy Eating Index 2010	283	0.98 (0.97–0.995)	0.0029	1.00 (0.99–1.02)	0.5470
Year of diagnosis	295	1.04 (1.01–1.07)	0.0056	0.98 (0.95–1.01)	0.2508
Conditions ^c^					
No	252	1.00	0.4104		
Yes	43	1.15 (0.83–1.60)			

^a^ HR model adjusting for age. ^b^ HR model adjusting for age, race, underlying etiology, stage, treatment, smoking status, education, BMI, Health Eating Index-2010, and year of diagnosis. ^c^ The presence of ascites, esophageal varices, or hepatic encephalopathy.

## Data Availability

De-identified dataset is available upon request.

## Supplementary Materials

The following are available online at https://www.mdpi.com/article/10.3390/cancers13143476/s1, Table S1: Characteristics of excluded HCC in the Multiethnic Cohort by race/ethnicity. Table S2: Association of race/ethnicity and other factors with HCC-related mortality. Figure S1: Overall (all-cause mortality) survival by underlying etiology (Log-rank *p*-value = 0.0019).
