# Supplementary material for: Etiology and Outcomes of Hepatocellular Carcinoma in an Ethnically Diverse Population: The Multiethnic Cohort
Source: Cancers (Basel). 2021 Jul 12;13(14):3476. doi: 10.3390/cancers13143476 (PMC8305188; doi:10.3390/cancers13143476)
Supplement: Supplementary file 1 [file cancers-13-03476-s001.zip › cancers-1269090-supplementary.pdf]

Figure S1. Overall (all-cause mortality) survival by underlying etiology (Log-rank P-value = 0.0019)
Abbreviations: ALD=alcohol-related disease; HBV=hepatitis B virus; HCV= hepatitis C virus; NAFLD=non-alcoholic fatty liver disease.

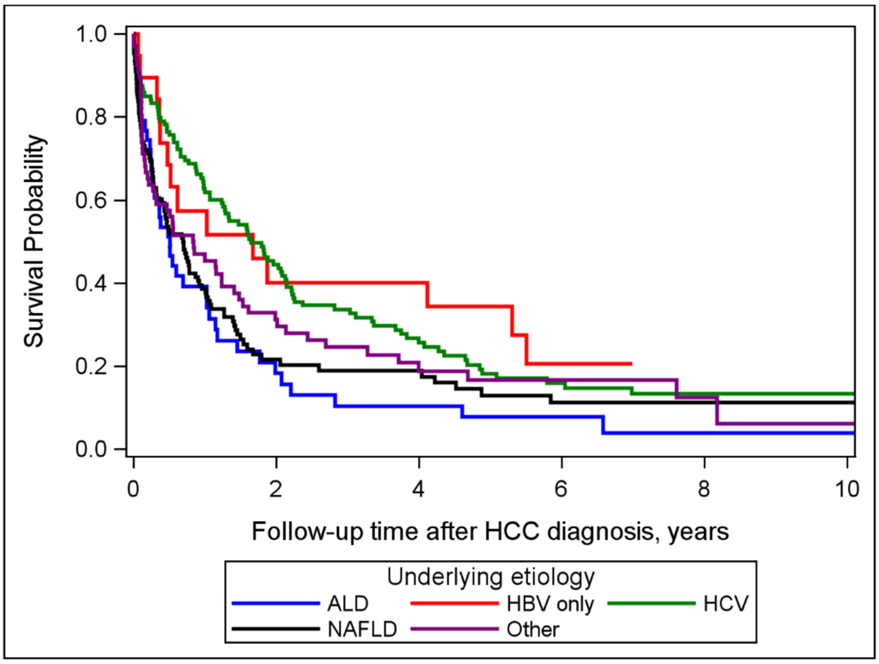

Table S1. Characteristics of excluded HCC in the Multiethnic Cohort by race/ethnicity

Characteristics	All Patients (N = 383)	African American (N = 60)	Japanese American (N = 105)	Latino (N = 143)	White (N = 46)	Native Hawaiian (N = 29)
Mean age at enrollment (SD), years	62.0 ± 8.17	61.1 ± 8.95	63.5 ± 8.30	62.2 ± 7.22	59.1 ± 8.39	62.2 ± 9.17
Mean age at diagnosis (SD), years	71.8 ± 8.97	69.7 ± 9.16	73.5 ± 8.78	72.3 ± 8.49	68.9 ± 9.17	71.9 ± 9.85
**Sex (%)**						
Male	68.2	68.3	61.9	67.1	80.4	75.9
Female	31.9	31.7	38.1	32.9	19.6	24.1
**BMI Category (%)**						
< 25.0	28.2	33.3	40.0	20.3	21.7	24.1
25.0 – 30.0	43.9	30.0	46.7	49.7	41.3	37.9
≥ 30.0	26.4	28.3	13.3	29.4	37.0	37.9
Unknown	1.6	8.3	0.0	0.7	0.0	0.0
**Smoking status (%)**						
Never Smoker	26.6	11.7	27.6	32.2	28.3	24.1
Ever Smoker	71.5	85.0	70.5	65.7	71.7	75.9
Unknown	1.8	3.3	1.9	2.1	0.0	0.0
**Education (%)**						
High school graduate or less	58.5	46.7	55.2	71.3	32.6	72.4
Some college or technical school	24.5	35.0	24.8	17.5	34.8	20.7
College graduate	8.6	6.7	10.5	6.3	15.2	6.9
Graduated and professional school	6.8	8.3	8.6	2.8	17.4	0.0
Unknown	1.6	3.3	1.0	2.1	0.0	0.0
**Stage at diagnosis (%)**						
Localized	40.0	36.7	48.6	38.5	30.4	37.9
Regional	20.4	20.0	19.1	21.7	21.7	17.2
Distant	24.3	26.7	21.9	23.8	26.1	27.6
Unknown	15.4	16.7	10.5	16.1	21.7	17.2
P-value*	0.8388	0.8746	0.1261	0.7225	**Reference**	0.8791
**First course of treatment (%)**						
None	53.5	71.7	39.1	57.3	52.2	51.7
Treated	42.6	25.0	58.1	37.1	47.8	41.4
Unknown	3.9	3.3	2.9	5.6	0.0	6.9
P-value*	0.0026	0.0182	0.2158	0.1530	**Reference**	0.2182

*For all patients, P values for differences by race/ethnicity. For each ethnic group, P values for comparing each group to whites. P values from Chi-square or Fisher's Exact when cells have expected counts less than 5.

Table S2. Association of race/ethnicity and other factors with HCC-related mortality

	No. deaths	Univariate HR (95% CI) [a]	P value	Multivariate HR (95% CI) [b]	P value
Race/ethnicity					
White	25	1.00	< 0.0001	1.00	0.0031
African American	28	1.82 (1.05-3.16)		2.66 (1.32-5.34)	
Japanese American	77	0.88 (0.46-1.68)		1.02 (0.59-1.74)	
Latino	68	0.91 (0.58-1.44)		1.53 (0.84-2.77)	
Native Hawaiian	15	1.91 (1.20-3.04)		0.75 (0.36-1.60)	
Sex					
Female	139	1.00	0.4189		
Male	74	1.13 (0.84-1.51)			
Underlying Etiology					
HCV	62	1.00	0.0003	1.00	0.0007
HBV	9	0.87 (0.43-1.77)		2.17 (0.96-4.90)	
NAFLD	69	1.87 (1.30-2.68)		2.61 (1.68-4.06)	
ALD	29	2.44 (1.54-3.84)		2.34 (1.36-4.02)	
Other	44	1.49 (1.01-2.21)		1.89 (1.16-3.07)	
Stage					
Localized	74	1.00	< 0.0001	1.00	< 0.0001
Regional	58	2.81 (1.98-3.98)		2.47 (1.69-3.61)	
Distant	45	5.02 (3.43-7.35)		4.71 (3.07-7.25)	
Unknown	36	6.76 (4.45-10.3)		2.63 (1.52-4.54)	
Treatment					
None	84	1.00	< 0.0001	1.00	< 0.0001
Treated	113	0.16 (0.12-0.21)		0.21 (0.15-0.31)	
Unknown	16	1.27 (0.70-2.31)		0.98 (0.47-2.03)	
Smoking status					
Never Smoker	58	1.00	0.1979		
Ever Smoker	150	1.32 (0.97-1.81)			
Unknown	5	1.39 (0.55-3.51)			
Education					
High school graduate or less	108	1.00	0.0343	1.00	0.1961
Some college/technical school	64	0.82 (0.60-1.12)		0.69 (0.48-1.00)	
College graduate	20	0.98 (0.60-1.60)		1.16 (0.66-2.06)	
Graduate/professional school	16	0.43 (0.25-0.73)		0.69 (0.37-1.30)	
Unknown	5	1.06 (0.42-2.64)		1.25 (0.39-4.04)	
Alcohol Intake					
0	103	1.00	0.1700		
< 12 g/day	52	1.22 (0.87-1.72)			
≥ 12 g/day	48	0.98 (0.70-1.39)			
Unknown	10	1.96 (1.00-3.85)			
BMI Category (kg/m^2)					
< 25.0	61	1.00	< 0.0001	1.00	0.0080
25.0 – 30.0	92	1.53 (1.11-2.13)		0.97 (0.66-1.43)	
≥ 30.0	58	2.62 (1.80-3.80)		1.88 (1.19-2.99)	
Unknown	2	1.31 (0.30-5.62)		0.60 (0.12-2.93)	
Diabetes					
No	81	1.00	0.5572		
Yes	132	1.09 (0.82-1.44)			
Healthy Eating Index 2010	203	0.99 (0.98-0.999)	0.0352	1.01 (0.99-1.02)	0.4215

Year of diagnosis	213	1.06 (1.02-1.09)	0.0011	0.99 (0.96-1.03)	0.7219

[a] HR model adjusting for age

[b] HR model adjusting for age, race, underlying etiology, stage, treatment, education, BMI, Health Eating Index 2010 and year of diagnosis
